# Supplementary material for: Individual prevention and containment measures in schools in Catalonia, Spain, and community transmission of SARS-CoV-2 after school re-opening
Source: PLoS One. 2022 Feb 16;17(2):e0263741. doi: 10.1371/journal.pone.0263741 (PMC8849486; doi:10.1371/journal.pone.0263741)
Supplement: S1 Table — (DOCX) [file pone.0263741.s003.docx]

**Supplementary Table 1:**

| **Total cases** | **Secondary cases** | **Frequency in primary schools** | **Frequency in secondary schools** |
| --- | --- | --- | --- |
| 1 | 0 | 0.786 | 0.712 |
| 2 | 1 | 0.141 | 0.172 |
| 3 | 2 | 0.0416 | 0.060 |
| 4 | 3 | 0.0154 | 0.0224 |
| 5 | 4 | 0.0076 | 0.0125 |
| 6 | 5 | 0.0024 | 0.0074 |
| 7 | 6 | 0.0021 | 0.0032 |
| 8 | 7 | 0.0013 | 0.0039 |
| 9 | 8 | 0.0006 | 0.0016 |
| 10 | 9 | 0.0009 | 0.0009 |
| 11 | 10 | 0 | 0.0013 |
| 12 | 11 | 0.0001 | 0.0003 |
| 13 | 12 | 0 | 0.0007 |
| 14 | 13 | 0 | 0 |
| 15 | 14 | 0.0001 | 0.0001 |
| 16 | 15 | 0 | 0.0001 |
| 17 | 16 | 0 | 0 |
| 18 | 17 | 0 | 0.0001 |
| 19 | 18 | 0 | 0 |
| 20 | 19 | 0 | 0 |
| **R*** |  | **0.35** | **0.55** |
