# Supplementary material for: Individual prevention and containment measures in schools in Catalonia, Spain, and community transmission of SARS-CoV-2 after school re-opening
Source: PLoS One. 2022 Feb 16;17(2):e0263741. doi: 10.1371/journal.pone.0263741 (PMC8849486; doi:10.1371/journal.pone.0263741)
Supplement: S1 File — (DOCX) [file pone.0263741.s004.docx]

**SUPPLEMENTARY MATERIALS AND METHODS**

**Computational model**

We developed a stochastic computational model for being used as a platform for virtual experiments. In the model, we consider *N* students per bubble in a school with N_c_ groups. As for initial conditions, we evaluate the probability to have an infected student by assuming the same incidence of the general neighborhood or the city. In particular, we set an initial N_init_ students taken from a random distribution according to this incidence. Once the simulation is running, more students can be infected by SARS-CoV-2. We consider the following two infection pathways:

*External infection*

Any student can be infected outside of the school with a probability proportional to the incidence, which we consider constant during the simulation. Therefore, we assume a stationary situation in the city (i.e., an epidemiological dynamic with an average value of R_t_=1).

*Internal infection*

Any student can be infected inside the school by an infectious pupil or member of staff of the same bubble. In order to model internal infection, we assume that the infectious agent can generate new infections both inside and outside the school (family or others). Assuming that roughly 70% of the time the student is outside the school, we consider that only 30% of his/her close contacts are inside the school (figure 1). We use this ratio to determine the probability of a kid to infect a classmate in the school bubble, given a certain transmission level.

The probability of contagion depends on the cycle of the infection. It rapidly increases at the beginning of the cycle, reaching the peak at 4-6 days post-infection, and then decreases with time. Some preliminary estimations for this probability density function are given in the article of McAloon *et al*^1^. According to this paper, there is an important decrease after day 8 of infection. We have considered the curve shown in Figure 1B for the probability density function, which is in consensus with the one discussed in the aforementioned paper and corresponds to a Gompertz-like function.


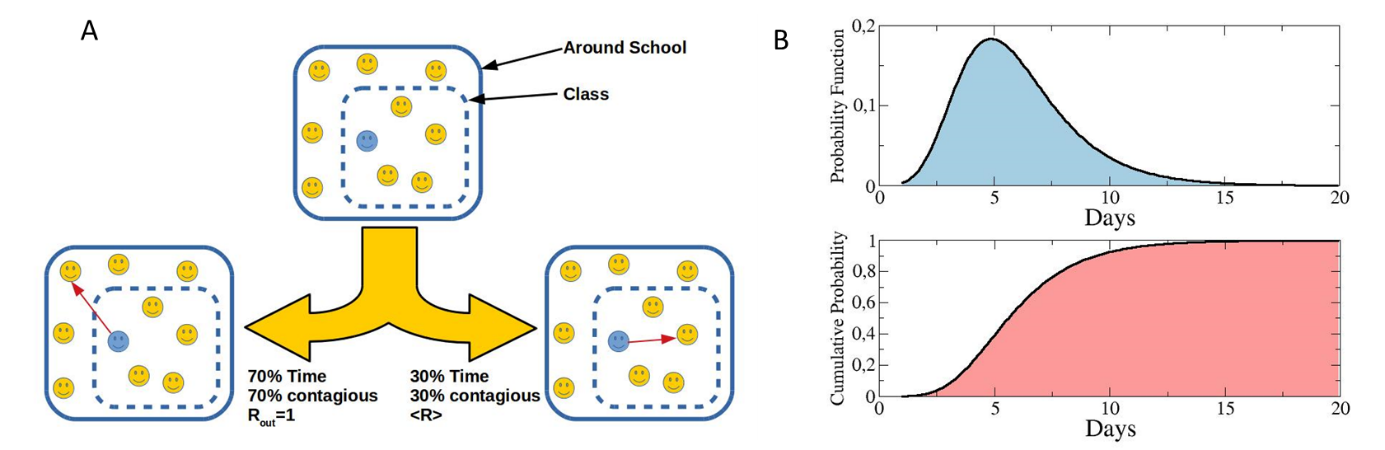


**Figure 1. Model summary.** (A) Structure of the contagion pattern, accounting for the transmission from an infected agent (blue) to an external contact (left arrow) or an internal classmate (right arrow), (B) Distribution of the probability of contagion use in the model. Variation on time of the normalized probability density function for the contagion (up, blue curve) and the corresponding cumulative probability function (down, red curve) employed in the mathematical model.

The model assumes that infection only happens when two individuals spend some critical time together and that the individuals spend packets of time with other individuals, assuming that interaction is restricted to only two individuals during such packet of time.

**Case detection and quarantining**

At the 5^th^ day of the infection, we evaluate if a student shows symptoms (30% of probability). In such case, we assume that his infection is detected, and we remove the individual (i.e., we do not permit more infections). In case the infected kid is asymptomatic, we evaluate a certain probability that the kid is detected by contact tracing or screening and we remove the individual. Globally, we assume that 70% of the infected kids are detected.

Each individual has a particular probability to infect other individuals that depends on their social behavior and physiological characteristics. As a result, they can infect more or less people. We assume that each infected individual can infect between 0 and R_max_ contacts, the precise value being randomly chosen between both. Therefore, the average propagation in a population is R_max_/2.

**Simulation scheduling**

The model incorporates a high level of stochasticity. Therefore, for each of the scenarios simulated, we ran 20 simulations with the same set of parameters and initial conditions. A total of 72000 bubble groups distributed in 5,130 identical schools were considered, therefore in each school there was a set of independent 14 bubble groups with 20 students each. Such distribution of groups in schools roughly accounts for the total number of students (~1.5M) accounted for by the Catalonian Educational system,

Numerical simulations take an aleatory flux of external infected students from outside of the school with a constant probability based on the external incidence. Inside the groups there is a contagion with a certain probability given by the probability of contagion R which is randomly selected from a homogenized distribution of values. As a first approach there is no interaction among bubble groups in the model. Results shown here for schools are given as the average of the 14 groups and the global quantities are given for 50 days simulations

**REFERENCES SUPPLEMENTAL MATERIAL**

^1^ McAloon C, Collins Á, Hunt K, et al. Incubation period of COVID-19: a rapid systematic review and meta-analysis of observational research. BMJ open 2020; 10(8): e039652.
