# Supplementary material for: Individual prevention and containment measures in schools in Catalonia, Spain, and community transmission of SARS-CoV-2 after school re-opening
Source: PLoS One. 2022 Feb 16;17(2):e0263741. doi: 10.1371/journal.pone.0263741 (PMC8849486; doi:10.1371/journal.pone.0263741)
Supplement: Attachment — Submitted filename: ResponsetoReviewers_04.10.2021.docx [file pone.0263741.s005.docx]

**Responses to reviewers**

**PONE-D-21-21706**

**Manuscript title**: "Individual prevention and containment measures in schools in Catalonia, Spain, and impact of this strategy on community transmission of SARS-CoV-2 after school re-opening"

**EDITORIAL COMMENTS**

1. Please ensure that your manuscript meets PLOS ONE's style requirements, including those for file naming. The PLOS ONE style templates can be found at

<https://journals.plos.org/plosone/s/file?id=wjVg/PLOSOne_formatting_sample_main_body.pdf>

and

<https://journals.plos.org/plosone/s/file?id=ba62/PLOSOne_formatting_sample_title_authors_affiliations.pdf>

**Author’s response:** Done. Files also renamed as suggested.

2. In your Data Availability statement, you have not specified where the minimal data set underlying the results described in your manuscript can be found. PLOS defines a study's minimal data set as the underlying data used to reach the conclusions drawn in the manuscript and any additional data required to replicate the reported study findings in their entirety. All PLOS journals require that the minimal data set be made fully available. For more information about our data policy, please see <http://journals.plos.org/plosone/s/data-availability>.

Upon re-submitting your revised manuscript, please upload your study’s minimal underlying data set as either Supporting Information files or to a stable, public repository and include the relevant URLs, DOIs, or accession numbers within your revised cover letter. For a list of acceptable repositories, please see [http://journals.plos.org/plosone/s/data-availability#loc-recommended-repositories](../../../D:/Dropbox/Recerca/CORONAVIRUS_ALL/Coronavirus/Articles_i_congressos/Plos_One_Escoles/Revisió/_blank). Any potentially identifying patient information must be fully anonymized.

Important: If there are ethical or legal restrictions to sharing your data publicly, please explain these restrictions in detail. Please see our guidelines for more information on what we consider unacceptable restrictions to publicly sharing data: [http://journals.plos.org/plosone/s/data-availability#loc-unacceptable-data-access-restrictions](../../../D:/Dropbox/Recerca/CORONAVIRUS_ALL/Coronavirus/Articles_i_congressos/Plos_One_Escoles/Revisió/_blank). Note that it is not acceptable for the authors to be the sole named individuals responsible for ensuring data access.

We will update your Data Availability statement to reflect the information you provide in your cover letter.

**Author’s response:** We have Table 1 with some data employed in this work. Such data have been obtained from private anonymized data from the education Ministry of Catalonia. Data about confined groups were obtained directly from Traçacovid:

https://analisi.transparenciacatalunya.cat/en/Educaci-/Dades-COVID-19-als-centres-educatius/fk8v-uqfv

The data organized by age and positivity are also public and can be found in:

https://dadescovid.cat/descarregues?lang=eng https://dadescovid.cat/static/csv/catalunya_diari.zip

We have incorporated such links into the manuscript.

3. Your ethics statement should only appear in the Methods section of your manuscript. If your ethics statement is written in any section besides the Methods, please move it to the Methods section and delete it from any other section. Please ensure that your ethics statement is included in your manuscript, as the ethics statement entered into the online submission form will not be published alongside your manuscript.

**Author’s response:** Done. The ethics statement was already included in the Methods section, but has now been expanded.

4. Thank you for stating the following in the Funding Section of your manuscript:
“Funding

The components of the analysis drawn from the different KIDS Corona platform have been funded by Stavros Niarchos Foundation (SNF), Banco Santander and other private donors of Kidscorona. We also acknowledge funding from La Caixa Foundation (ID 100010434), under agreement LCF/PR/GN17/50300003; and funding from Ministerio de Ciencia, Innovación y Universidades and FEDER, with the project PGC2018-095456-B-I00. This work has been also partially funded by the European Commission - DG Communications Networks, Content and Technology through the contract LC-01485746. ISGlobal receives support from the Spanish Ministry of Science and Innovation through the “Centro de Excelencia Severo Ochoa 2019-2023” Program (CEX2018-000806-S), and support from the Generalitat de Catalunya through the CERCA Program. CISM is supported by the Government of Mozambique and the Spanish Agency for International Development (AECID). NB is supported by an FPU predoctoral fellowship from the Spanish Ministry of Universities (FPU18/04260). BB is a Beatriu de Pinós postdoctoral fellow granted by the Government of Catalonia’s Secretariat for Universities and Research, and by Marie Sklodowska-Curie Actions COFUND Programme (BP3, 801370).

Role of the funding source

The funders had no role in the interpretation of the data or in the writing up of the manuscript.”

Funding information should not appear in the Funding section or other areas of your manuscript. We will only publish funding information present in the Funding Statement section of the online submission form.

**Author’s response:** This has been included in the Funding section of the manuscript, at the end of the text, under the “Declarations” section.

_____________________________________________________________________________

REVIEWER COMMENTS

**Reviewer #1**:

The authors tackle an important health issue in whether schools should stay open or close due to COVID-19. They have access to a unique dataset that purports the number of secondary infections in schools in Catalonia. They then constructed models to estimate the transmission within schools and how school aged children contributed to the overall transmission of COVID-19 in Catalonia.
I have listed the comments in chronological order of the manuscript.

**Author’s response:** We thank the referee for his/her comments. In our answers we will often refer to the publication “Age-dependency of the Propagation Rate of Coronavirus Disease 2019 Inside School Bubble Groups in Catalonia, Spain” (doi: 10.1097/INF.0000000000003279). In this paper, that was published this summer, we report the experimental evaluation of R* in Catalan schools during the first term. These experimental data were not available when this manuscript was prepared, and we based our hypothesized values on the measurements of the transmission in Catalan summer schools (doi: 10.1093/cid/ciab227). Right now, we can affirm that the experimentally measured transmission in Catalan schools is in the range that we assumed for the modelling approach. Therefore, this reinforces the validity of our approach.

Lines 283-3 pg 11. The authors mention that the global detection rate of COVID-19 in children is 70%. How did the authors choose this number? Is it based on the literature? If so, please cite?

**Author’s response:** In the manuscript “Asymptomatic SARS-CoV-2 infection: A systematic review and meta-analysis (<https://doi.org/10.1073/pnas.2109229118>) states that asymptomatic population is around 35% of the cases, therefore if the infection of the children is inside the house from the parent 65% of relatives were symptomatic and likely to be detected. The rest of cases are children coming from an asymptomatic infection (0.35%) and the probability of children to be asymptomatic is higher 50% see “Household Severe Acute Respiratory Syndrome Coronavirus 2 Transmission and Children: A Network Prospective Study” (<https://doi.org/10.1093/cid/ciab228>) and the probability of detection is 80%. However there are also infections inside the school with an average of 50% of probability to be detected. We have chosen an average approximated value of 70%.

We have incorporated such estimations in the corresponding paragraph in the manuscript.

Lines 286-7 pg 12. Can the authors explain why they chose to keep community transmission at R = 1? At such a low R would there even be a debate about whether or not to close schools? Given the access to data the authors had, couldn’t they have estimated R in the community during the time of their simulation and made R variable based on estimated actual conditions?
**Author’s response:** The value R=1 is not employed to simulate the reality outside the schools. The reason is to maintain the conditions of our simulations under control. As we have found in “Age-dependency of the Propagation Rate of Coronavirus Disease 2019 Inside School Bubble Groups in Catalonia, Spain” (doi: 10.1097/INF.0000000000003279) and it is here confirmed is that although the entrance of index cases does depend on the incidence outside the schools (one can assume it is proportional) the propagation rate inside the bubble groups is independent of such incidence outside the schools. Therefore, to study the propagation we fix the rest of conditions and parameters and vary only the propagation (R*) inside the school.

Lines 305 pg 12. The authors estimate that children spend 30% of their time in school and thus the R in school is 30% of the total R. Can the authors clarify how they calculated 30%? First off, does this fraction take into account weekends? Also, have the authors taken into account that ~33% of the day that children are asleep and are highly unlikely to infect anyone if they sleep alone and can probably only infect whoever they share a room with? Would the fraction make more sense based on the number of waking hours they spend in school?
**Author’s response:** The calculation of R* inside the school was done with the time spent in the school in comparison with the time expended outside the school considering weekends and only waking hours, as noted actually by the reviewer.

However, we admit that the choice of a particular value may be confusing and therefore we have employed in the new version of the manuscript the propagation inside the schools and compare only with the value inside the schools.

Lines 339-341 pg 13. The authors state that they evaluated four scenarios but list five different size bubble groups. Is this a typo or am I missing what the four scenarios are?
**Author’s response:** We thank the reviewer to note this typo, yes there were five different scenarios and we have corrected the new version of the manuscript.

Lines 348-9 pg 14. The authors state that they are evaluating R* from 0.3 - 1.5, which translates to an R of 1 – 5. Can the authors comment on why they chose to study the range of R values they did? Are these in line with the range in R that was calculated in Catalonia during the study period? Also, it may be helpful to include both R* and R in the figure legends to give the numbers more context.
**Author’s response:** In the new version of the manuscript, we have removed the equivalence between R and R* to avoid misunderstandings. The values of R* between 0.3 and 1.5 were considered to account to different possible conditions where schools did not give rise to large propagation (R* << 1) and conditions where the schools trigger the propagation (R*~>1). Actually, we can compare with our recent study (doi: 10.1097/INF.0000000000003279), where R* was found to be between 0.2 and 0.6.

Figures 1 and 2. The authors state that the model is stochastic and that they have multiple runs, yet the plot is represented as a bar plot. Did all runs produce the exact same number of secondary infections? If not, can the authors please replot and replace the bar plots with boxplots to show how much variation there is in the model?
**Author’s response:** The variation of the results obtained are small because the number of bubble groups considered inside the schools is very large, giving rise to quite homogenized values of number of cases. We have, however, included the standard deviations in the representation of the data to account for the dispersion.

Figures 1 and 2. While the number of secondary infections is interesting, it might be more useful to show the number of secondary infections per 10,000 students in order to give a larger context on the severity of the spread of COVID-19. While the authors do provide the number of students in the figure caption plotting rates on the y-axis would make it easier to evaluate the impact of secondary infections according to the model.
**Author’s response:** We have changed the y-axis from total number to secondary infections per 10,000 students as proposed by the reviewer.

Lines 365-371 pg 14. The authors have great data on the impact of bubble groups in Catalonia. Do the authors by any chance have the distribution in sizes of the bubble groups (i.e., what percentage were of size 20)? Also, how did the authors determine that the size of the bubble groups is most likely 20?

**Author’s response:** Such information would be great to have and to be able to use, unfortunately, we do not have access to such information.

Line 375 pg 14. The authors state “a statistically significant difference…” Unlike many manuscripts this manuscript does not rely heavily on results driven by p-values and I commend the authors for this effort. I believe in this instance that the authors are referring to the results of another manuscript, but would appreciate if they removed “statistically significant” from this manuscript. Perhaps they could simply delete “statistically significant” and leave “a difference…” The reason for this comment is that the statistics community has recently come out strongly against p < 0.05 and away from using the terms statistical significance, which to their community is not a term that has meaning (please see Wasserstein et al. 2019 <https://doi.org/10.1080/00031305.2019.1583913> ; also for a brief history of p-values see Nuzzo 2014 - <https://www.nature.com/articles/506150a>). I understand that such a transition will take time but would encourage the authors to change their language in order to help promote better science. Thank you in advance.
**Author’s response:** We thank the reviewer, and we completely agree. We have removed this terminology as suggested.

Line 385 pg 15. The authors talk about incidence accumulated over 10 days (A10). Can the authors please clarify if this the cumulative incidence reported on the date in question or is it the actual incidence for the 10 days prior to the date in question? i.e., could this model be used to predict based on the current known data? Or is calibrated to modeling weeks or months after the fact?
**Author’s response:** The incidence accumulated over the last 14 days or over the last 7 days are usual indexes employed during the pandemic to evaluate the situation. Here, we have employed the cumulative incidence over the last 10 days to compare with the number of confined groups, which are closed when a positive appears (corresponding to one of the cases determining the incidence) and remains closed for 10 days of quarantine.

Lines 389-395 pg 15. The authors mention uncertainty in the effective time spent in school and the probability of detection. Have the authors tried using their model to search this parameter space to get a better sense of the most likely percent of effective times spent at school and the percent probability of detection? Getting a sense of the range and likelihood of these values would be an important contribution to informing whether schools should open.
**Author’s response:** This is an interesting point. However, the number of confined groups would depend on the combination of both factors and therefore it is difficult to obtain one of the parameters without the effect of the other from the data in schools.

Line 407 pg 15. There is an empty box followed by a * instead of a symbol. I imagine this was some sort of file conversion error and wanted to give you a heads up.
**Author’s response:** We thank the reviewer for spotting this formatting error, which has been now corrected.

Lines 427-432 pg 16 – Supplementary Table 1. Can the authors please clarify the cases column in supplementary table 1. It looks as if that for every primary infection there is at least one secondary infection. Perhaps shift the scale to start at 0 and rename secondary infections.
**Author’s response:** We have noted the table as cases precisely to indicate that it does not correspond to secondary cases. However, as it may be misunderstanding as stated by the reviewer, we have added a new column with the secondary cases corresponding to (cases - 1)

Figures 4 A and B. Again, I believe that “Number of Infected Kids” is meant to show the number of secondary infections where 1 means there were no secondary infections. I believe it would be easier to understand these plots if the scale was shifted and the x-axis was renamed Number of Secondary Infections. Also, since the y-axis is log-scale and the first column is near 0.7 or 0.9 it might be helpful to include minor gridlines to better see the difference, which looks small on log scale but is quite large in terms of this study.
**Author’s response:** As proposed by the reviewer we have shifted the data in the x-axis and renamed the axis. We agree that the values of the first column are very important and that differences between 0.7 and 0.9 are relevant. Actually, therefore we have figure 4C (new fig 4E). We have added a sentence in the text explaining that

Lines 437-8 pg 16. Can the authors comment on how the results of the model would change if the R outside of schools > 1? Wouldn't this be worth exploring since often the biggest driver of people wanting to shut down schools is when R > 1 in the community.
**Author’s response:** The propagation rate inside the school is independent on the number of kids infected outside because of an increasing of the surrounding incidence (changing R outside). Actually, we mean with R=1 in the text a continuous influx of infected students inside the bubble groups. Only for very high incidences it may affect the probability to double entrance of cases, very rare for small incidences. We are not trying to mimic the entrance of infected children in the schools but the propagation inside.

Lines 438-9 pg 16. The authors state that the reason they did not run R* = 0 but did not state why they choose R* = 0.15 – 0.9. Can the authors comment on why they chose to model this range of R* values?

**Author’s response:** It is related with the observed values published in our recent study (doi: 10.1097/INF.0000000000003279) and with the values which may fit curves in Fig.4A-B. See also new panels C and D on figure 4.

Lines 440-2 pg 16. The authors state that their model fits the data from the schools. Can the authors state what are the predicted R*s for each school? How did the authors determine which R* was the better fit (i.e., what measurement did you calculate to determine the best fit)?
**Author’s response:** The typical values of R* have been experimentally determined in “Age-dependency of the Propagation Rate of Coronavirus Disease 2019 Inside School Bubble Groups in Catalonia, Spain” (doi: 10.1097/INF.0000000000003279) to be between 0.2 and 0.6 and therefore we employ such values as predicted for each school. Furthermore from Figure 4 we can obtain such values from two sets of data, for primary and secondary schools: R*=0.35 and R*=0.55, respectively. We have compared in the new figure of the supplemental material / new panel on Figure 4 the mean square displacement between the results from the simulations and the distribution observed in the school and the minimal error corresponds roughly to values of the denoted values 0.35 and 0.55).

Figures 4C and 4D. Please add figure legends.

**Author’s response:** We have added the figure legends in the Figures 4E and F.

Line 460 pg 17. Small typo. The authors wrote “stablished” when I believe they meant to write “established.”
**Author’s response:** This has been corrected.

Figure 5. Please add a colorbar.

**Author’s response:** A colorbar has been added as a legend.

Lines 517-20 pg 19. I love the point the authors make regarding schools only having a slight contribution to overall transmission. That being said the results only show schools having a slight contribution with the variants of COVID-19 studied. New variants such as B1.617.2 are more transmissible. Have the authors looked into incorporating a more transmissible variant into their model and how much schools could contribute with said variant? Also, can the authors quantify how much schools contribute to overall transmission (e.g., 10% to overall transmission or 20% less than expected)?

**Author’s response:** In our opinion the most important result is the low value of R* which is actually able to reproduce the data. It indicates that schools do not amplify the propagation. The transmission inside schools in comparison with the overall transmission is small as experimentally assessed in “Age-dependency of the Propagation Rate of Coronavirus Disease 2019 Inside School Bubble Groups in Catalonia, Spain” (doi: 10.1097/INF.0000000000003279)

Lines 547-8 pg 20. The authors state “but our data suggest that transmission among the youngest remains minor, even in the absence of facemasks.” How do the authors justify this statement? From what they state the models are based on school data where students were required to wear masks. Please justify or remove.

**Author’s response:** In Spanish schools, the use of facemasks is only compulsory for those children aged 6 and above. The data that we have from the different schools also includes children younger than 6 years of age, and thus not wearing masks. We have just clarified this in the text.

Line 551 pg 20. Why do the authors mention B1.1.7 but do not mention the more prevalent and more transmissible B1.617.2? Also, these variant names may be a bit too jargony for this manuscript. Perhaps replace or add alpha and/or delta.
**Author’s response:** The reason for not mentioning variant B1.617.2 is that at the time the manuscript was written, this variant was not yet of concern nor had it spread. Unfortunately, this manuscript circulated during a long time before it could be efficiently reviewed, and that explains why some of the information is not updated. We have now amended this.

Lines 581-3 pg 21. The authors state “Closing schools should only be considered in the worst-case scenario, where all other possible transmission containment measures have been adequately implemented.” Based on this study could the authors state what a worst-case scenario would look like. Also, can this model be used to advise schools when they are approaching a worst-case scenario?

**Author’s response:** It is not easy to define what can be considered worst-case scenario, however, we believe that when incidence rates are on the rise, the health system is at risk of collapsing, and the general opinion is that a full home confinement may be warranted, then, it may be the moment where schools also have to close. However, if only certain activities are interrupted, then schools should not be among the first things to close.

The authors show that having schools open did not greatly impact the greater community and also did not lead to many known secondary infections in school, which I find to be one of the most interesting findings in this manuscript. However, we originally shut down schools during this pandemic because we looked at how important schools were in the transmission of diseases during previous pandemics and epidemics. Therefore, we should be cautious in implying that because past school closures due to COVID-19 were not necessary to limit transmission because that does not necessarily mean that future schools closures due to COVID-19 are not necessary given the increased transmissibility of new variants.

**Author’s response:** The reviewer is completely right, and this nuance has been added to our conclusion.

_____________________________________________________________________________

**Reviewer #2**

The manuscript is devoted to the analysis of the 2020 Autumn term of the academic year in Catalonia Schools using a public database of infections in Schools.

Although there is no clear goal or main outcome of the paper, the main points of the manuscript are the following:

(G1) The authors do have an opinion on the importance of presential school, which I think would be more suitable for an opinion letter to a scientific journal.

(G2) The authors describe some aspects on the number of students infected, classes confined and number of secondary cases per infected student.

(G3) The authors present a 'modeling exercise' (in their own words) to "demonstrate the limited role of childhood transmission in relation to overall community transmission when proper epidemiological surveillance and recommendations are in place." To this end, their intention is to compare the output of their simulations with data from schools and incidence in Catalonia during the same period.

**Author’s response:** We thank the reviewer for the insightful comments, but we respectfully disagree with some of them. The manuscript (and its underlying analysis) did have clear goals and outcomes. We indeed have an opinion on the importance of presential school, shared with the vast majority of pediatricians and educational experts. However, we do not think our opinion blurs our objective evaluation of the data presented. We have rewritten our affirmations about in-class school including some references which confirms the objective fact of low-propagation in schools and the higher infections probability from adult to children than from children to adults. With such objective facts, our study models the propagation inside the schools and try to estimate the incidence.

A web search of "SARS School Catalonia" reveals several other papers on this dataset that have not been commented on the present manuscript. The authors should compare their findings with existing analysis of the same dataset.

**Author’s response**: the reviewer is right in terms of other analyses regarding this (or similar) dataset. This paper was the first analysis conducted, but the manuscript suffered an ordeal ad remained in a journal “under review” for several months without actually reviews being provided. We were obliged to withdraw the manuscript and send it elsewhere given the inaction of the previous editors. In the meantime, we conducted further analyses (not duplicated, but rather complementary, and digging with more depth in certain aspects hinted at in this first manuscript). We had a quick review process with that second manuscript which was accepted much earlier than we received the reviews for the current one. We can certainly include a reference when we are discussing the values of R* to the published manuscript given that in that paper, we were able to stratify the evaluation of this parameter according to age group. A paragraph has been added in the discussion with the comparison with other publications about propagation inside schools in Catalonia.

#2. Results reported have not been published elsewhere.
NO.

The description of infections in classrooms (item (G2) above), for instance what they call the reproduction number or the number of primary cases which define no secondary infections, have been partly reported by some of the authors in the paper:

"Age-dependency of the Propagation Rate of Coronavirus Disease 2019 Inside School Bubble Groups in Catalonia, Spain". doi: 10.1097/INF.0000000000003279
**Author’s response:** See answer and explanation provided below

#3. Experiments, statistics, and other analyses are performed to a high technical standard and are described in sufficient detail.
NO

The manuscript compares data from the schools registry (item (G2) above) with the outcome of their simulations (item (G3) above).

There is no statistical framework which supports the comparison of data with the result of the simulation. The authors use the word "significant" without referring to a statistically significant test, inducing confusion.

**Author’s response:** We have removed the word “significant” wherever this term was not adequate and have added more details on the statistical framework.

We have employed the average value of secondary cases per index case (definition of R* value) obtained from the data in schools and analyzed in “Age-dependency of the Propagation Rate of Coronavirus Disease 2019 Inside School Bubble Groups in Catalonia, Spain” (doi: 10.1097/INF.0000000000003279) to obtain a value to compare the results of the simulations. The comparison has been done using the mean square displacement for the evaluation of the differences between the simulations and the data.

The simulations are a not presented in a standard fashion and their desription is insufficent, in my opinion. Many important parameters like Rmax or the probability distribution that a positive child is detected, for instance, are not properly described. In my opinion, the simulation cannot be reproduced. Given the many unknowns around the infection processs parameters, any simulation should include estimates of uncertainty or at least a thorough sensitivity analysis. Lack of code availabaility is also problematic given the computational approach.
**Author’s response:** We have worked on the manuscript and tried to improve the description and presentation of our simulations. We do hope that these are now more acceptable. Additionally, we have added the code employed in an open access repository: <https://github.com/BIOCOM-SC/>BubbleGroups.git

#4. Conclusions are presented in an appropriate fashion and are supported by the data.
NO

A major issue with this manuscript is the inclusion of several conclusions which have important implications either clinically or for policy makers and are not supported by the data nor the analysis.

(1) L 541-544: The authors state that "transmission seemed not to be significantly increased with larger bubble groups". There is no statistical test which could yield significance and the conclusion on the size of groups, which has strong policy implications, is not supported by results.

**Author’s response:** We have rephrased the sentence to make it clearer.

(2) L 544-546: "the ideal confinement duration of contacts was 10 days, with transmissibility enhanced with shorter timings, and no additional effect when expanding the duration to two weeks." or "Our models suggest that the optimal confinement time for the contact of a positive case is 10 days" (L 141-142). In fact, their assumption is that "the detected kid always stays at home time enough to recover at home and become non-infectious" (L291) which further limits their analysis.

**Author’s response:** There is an error on the last sentence, in our simulations we actually include the possibility that the students can infect after the quarantine although the probability of infection strongly decreases with the time, in other case there will be no difference among the different times of quarantine. The sentence refers that the most usual situation is that after a quarantine of 10 and 14 days there are not more infections, basically because the use of the probability of infection shown in SFig1 is compatible with published probabilities. Therefore, the results do confirm that given such types of probabilities the quarantine of 10 days are comparable in effectivity with the quarantine of 14 days.

(3) L 546-548: "We did not assess the impact of mask wearing, which in our setting is only compulsory among pupils aged 6 or more, but our data suggest that transmission among the youngest remains minor, even in the absence of facemasks". This is not even discussed in the paper and the conclusion on the lack of effect of face masks contradicts recent CDC guidance that masks should be worn indoors by all individuals (age 2 and older).

<https://www.cdc.gov/coronavirus/2019-ncov/community/schools-childcare/k-12-guidance.html>

**Author’s response:** In Spain, the use of face masks is only compulsory for children aged 6 and above. We are aware of the CDC recommendations, although these have not been implemented in Spain. Indeed, the Spanish Pediatrics association has issued specific recommendations for the use of face masks, further supporting the age limit for compulsive use (indoors or outdoors) at 6 years of age. We of course here are not trying to assess and confirm/contradict the effects on transmission of wearing face masks. We only discuss our findings in the context of Catalan schools, whereby Catalan pupils (which include children younger and older than 6 years of age) have used (or not) facemasks according to their age. In any case, we have rephrased and toned down our discussion in this specific aspect.

(4) In "What do these findings mean?" it is stated that "School reopening (...) is safe for children and adult staff" although the only reference to adults in the paper is in the conclusions (lines 566-570) when they acknowledge the impossibility of deriving conclusions on the "adult component of the school inhabitants".

**Author’s response:** The reviewer is right, as we failed to include specific data on adult infections (among teachers and other adult staff). Having said so, the amount of infections among adults working in schools (<https://educacio.gencat.cat/es/actualitat/escolasegura/tracacovid/dades-covid19-centres/#/> ) has remained low, and other authors have also reported similar findings regarding adults working in schools. We have removed the reference in the “What do these findings mean” and added such reference (<https://pubmed.ncbi.nlm.nih.gov/33688033/>) to the conclusion component in the manuscript. However, the resulting propagation rates when the simulations are compared with the data from Catalan schools confirmed that propagation rates inside bubble groups are small and therefore school opening relatively safe, as it is stated in the ECDC.

(5) The authors state in the abstract that their results imply "decreased transmissibility for children in comparison to adults". I think this is not supported by the analysis presented.
**Author’s response:** We have toned down this statement, and removed the comparison to adults

The findings of the article are difficult to put in context because the authors use some non-standard terminology and definitions of important notions are lacking. We mention the following:

(1) OUTBREAK. There is no definition of "outbreak" (index case, follow-up period, etc...). Besides, there are contradictory statements on this concept:
- Abstract: "(...) scarce evidence of significant intraschool transmission in the form of outbreaks." (also in the methods section).
- lines 523-524 virtual absence of any remarkable child-driven outbreak reported (this conclusion is given without justification).
- Table S1 "Distribution of secondary cases per bubble groups in school outbreaks inside primary and secondary schools". This implies hundreds of outbreaks.
**Author’s response:** The reviewer is right that perhaps we have used “lightly” the concept of outbreak. We have now defined what we meant by “school outbreak” in the text.

(2) PRIMARY vs SECONDARY CASES: How are cases classified between primary and secondary?
**Author’s response:** Cases in the Primary school corresponds to students attending groups in the primary school in Catalonia, which are associated to students with ages typically between 6 and 11 years old. Cases in the Secondary school correspond to students attending groups in the primary school in Catalonia which are typically associated to students with ages between 12 and 15 years old.

(3) R and R*: One of the main outcomes of the manuscript is the "effective reproductive number" but no definition is given. Although the effective reproduction number may seem a simple concept its definition is tricky and has to take into account the epidemiology of the particular disease. The definition of R is referred to a preprint by some of the authors (which is not included in the references). Although the authors refer to the work of Li et al [24] on household transmission they do not use a similar approach. Besides, it is not correct to compare several values of the reproduction number (in "the community" or "schools) obtained from several definitions and to draw conclusions about them. The authors do not seem to use any standard technique to assess the value of R in the general population.
**Author’s response:** The referee is right; the estimation of the reproduction number is tricky. We have extended the definition of the methodology in the Methods section. We use an empirical estimation of R* that was adapted from Li et al [24] and already used in Jordan et al (<https://doi.org/10.1093/cid/ciab227>) and in Alonso et al (<https://doi.org/10.1097/inf.0000000000003279>), which assesses the average number of contagions per index case. In addition, Jordan et al proposes a way to compare the effective reproduction number with the transmission in the general population, taking in to account the time framework. Although this method is not exempt of bias, it can be considered as a good proxy. We have included it in the mentioned discussion.

(4) The authors define an indicator termed "homogenized cumulative incidence" (L252) which is a modification of the incidence. If the authors wish to use the indicator they define and draw conclusions, they need to properly analyze this quantity. This methodology would require an statistical analysis which is absent in this manuscript nor in the references they use.
**Author’s response:** This variable does not pretend to correct the incidence, as explained in the text, but to provide a simple way to take the diagnosis effort into account when trying to compare incidences between age groups that were submitted to different testing ratios. In any case, the discussion and conclusions we derive can be also obtained with supplementary figures 1 and 2 and are in concordance with: <https://doi.org/10.3389/fped.2021.754744>. The use of relative homogenized incidence facilitates this discussion, but it can be re-written in terms of SF1 and SF2, if necessary.

We have tried to better explain the meaning of this variable in the Methods section.

(5) Tests. Did the protocol use PCR-testing or other rapid tests? There is a contradiction also with mass screenings. L237: "Although originally planned, no mass screening and testing of teachers or pupils was conducted during the first trimester" but later in line 474: "Besides, several mass testing campaigns in the institutes within high incidence neighbourhoods were performed".

**Author’s response:** The protocol in place at schools used a variety of screening methods according to the knowledge of the virus that was progressively been acquired. Suspected cases were tested by means of nasopharyngeal or nasal PCR, and contacts of those cases were also subject to the same PCR screening. However, given the invasiveness of the method, unpleasant for children, some attempts were made to standardize salival PCR, but the use of this less invasive method was only applied in a few schools. Additionally, when antigen testing became available, many schools switched to this method, given the faster turnaround of results. Massive PCR screening was not systematically conducted in schools, although it may have been applied (in a limited way) in a few exceptional cases. We thank the reviewer for spotting the incoherence in our text and have amended the sentence which alluded to several mass testing campaigns.

#5. The article is presented in an intelligible fashion and is written in standard English.
NO

Althought the article is written in a correct standard English, the presentation suffers from several important problems. Apart from the absence of definition of the key objects presented in item #5 above, the structure and writing missess important aspects:

- Study period? There is no study period defined. I understand that it is more or less the Autumm term, but precise dates are missing and conflicting dates are given in different places of the paper. This happens, for instance, in lines 243, 275, 286 and 760.

**Author’s response:** We have now included more precise dates, so as to be more concrete about the study period.

- There is no clear IMRaD structure: results and conclusions are scattered all over the paper.

**Author’s response:** We respectfully disagree with this, and we think our manuscript has clear and well-defined Introduction, Methods, Results, and Discussion sections. It is true however, that many additional methods explanations and some results have been placed as supplementary material, but we think the rest of the manuscript is coherently presented.

- The manuscript needs to be updated (for instance, refers to B.1.1.7 as a potential threat). Updated systematic reviews should be included in the introduction. Some sentences in the introduction are stated without justification.

**Author’s response:** Thank you for these suggestions, which we have followed. We have included text on the Delta variant which had not reached Spain at the time we wrote the manuscript. We have added 4 new references in the introduction, all of them of updated systematic reviews.

#6. The research meets all applicable standards for the ethics of experimentation and research integrity.
NO

The ethics statement is confusing: it refers to "summer camps" and informed consent, but it is not clear which students or adults had an informed consent because no individual data from summer camps is used.
**Author’s response:** Thanks for spotting this mistake, which has now been corrected.

7. The article adheres to appropriate reporting guidelines and community standards for data availability.

NO

Although some data appears to be restricted for data protection, no adequate summary is presented (no table 1 with demographics description). Lack of code availability and diagnostic tests for the simulations limit the interpretation of the simulation.

**Author’s response:** Data sources have been further clarified, and the full data set has now been made available.

_____________________________________________________________________________

**Reviewer #3**:

This is an interesting manuscript aiming at studying the impact of the reopening of schools in the SARS-CoV-2 community transmission in Catalonia, Spain. Focusing on a children population is smart as it has not been well described in the literature and because school closure may have consequences on the kids' education and mental health. Results of the study showed that SARS-CoV-2 infections in schools mirror the community transmission. Closing schools should be the last resort option to limit the spread of SARS-CoV-2 when all other preventive measures have already been implemented.

Minor issues:
1. It would be interesting to discuss more about the vaccination against COVID-19 as children between 12-18 years old are eligible for being vaccinated and how it might impact the R* and therefore the transmission in schools and in the community.

**Author’s response:** We thank you for your suggestion. At the time the data were gathered, vaccines for teenagers had not yet been approved for their specific use in the 12—18 age group in Spain. Given that these are now widely available, we have added a sentence in the discussion mentioning vaccines as part of the strategy.

2. It is known that symptomatic persons are more likely to be able to spread the virus compared to asymptomatic persons. Is there a difference according to the presence of symptoms in terms of R* among children in schools?

**Author’s response:** No, we did not differentiate on propagation capacity between symptomatic and asymptomatic, it could be incorporate in the model, however it will demand the use of the new parameter with the relative difference. We do not expect substantial changes in the aggregate results.

3. Rounding decimals would improve the legibility of the Suppl Table 1.

**Author’s response:** This has been done. Numbers are now presented rounded to 3 decimals.

4. Suppl Table 1 appears twice in the document

**Author’s response:** We thank the reviewer and have tried to solve this. We believe it was due to the uploading system at the Journal.
