# Supplementary material for: Individual prevention and containment measures in schools in Catalonia, Spain, and community transmission of SARS-CoV-2 after school re-opening
Source: PLoS One. 2022 Feb 16;17(2):e0263741. doi: 10.1371/journal.pone.0263741 (PMC8849486; doi:10.1371/journal.pone.0263741)
Supplement: Attachment — Submitted filename: Response 2 toReviewers.docx [file pone.0263741.s006.docx]

**Second response to reviewers**

**PONE-D-21-21706**

**Manuscript title**: "Individual prevention and containment measures in schools in Catalonia, Spain, and impact of this strategy on community transmission of SARS-CoV-2 after school re-opening"

_____________________________________________________________________________

REVIEWER COMMENTS

**Reviewer #1**:

I appreciate the time and care the authors took in responding to the reviewer comments. I also empathize with the long review process the authors have experienced resulting in the delay of the dissemination of this work. The manuscript is much improved, but there are still a few minor comments to address.
Below are some general themes that I feel should be addressed.
Overall, a lot of work needs to go into the improvement of word choices and grammar (e.g., lines 178-9 “children were subject to the most severe of the lockdowns.”). Unfortunately, PLOS ONE does not copyedit, so I would suggest seeking help with the wording and grammar if necessary.

**Author’s response:** We thank the referee for his/her very constructive and encouraging comments. We have tried to review the word choices and grammar. In particular, we have amended the sentence in lines 178-9, as suggested.

Another general theme is putting this work in temporal context. Often the authors use present tense to describe the transmission of COVID-19 in schools. While I acknowledge that the present tense makes for better scientific writing, I am afraid that given the dynamic nature of this pandemic that readers may be under the impression that the results in this manuscript can be directly translated into the present reality of the pandemic which now has new variants and vaccines. I know the authors mention the variants and vaccines at the end of the manuscript, but I feel that an earlier emphasis on the fact that the results and conclusions of this study are based on Fall 2020 data would help prevent any confusion.

**Author’s response:** We have included a sentence at the beginning of the discussion emphasizing that the data are from the fall of 2020 and that things may have changed. We have reviewed the manuscript regarding the past/present tense, and have made sure that readers understand that the data are only valid for the period when they were collected.

I list more specific comments below in chronological order below.

In the conclusion of the abstract the authors state that “…cases detected in Catalan schools appear to closely mirror the underlying transmission from neighborhoods…”. This is a small, but I believe to be an important comment which is that more emphasis needs to be placed on the fact that this data and consequently the conclusions are tied to the COVID-19 pandemic in Fall 2020. I think it is important to clarify that any “mirroring” of underlying transmission and the success of preventative measures are all based on the pandemic pre-delta variant and pre-vaccinations. If the authors could add this disclaimer to the conclusion of the abstract I think it will help eliminate any confusion as to whether the same dynamics are driving the pandemic in the Fall of 2021. I would take the same approach to lines 147-149 under what did the researchers find? And under the section what do these findings mean? Lines 151-155 should also be qualified to acknowledge that the results in this manuscript and subsequent conclusions only apply to the COVID-19 pandemic pre-delta variant and pre-vaccines.

**Author’s response:** This has been amended as suggested.

Line 168 typo? I think the authors mean to write “containment” instead of “contention”

**Author’s response:** this has now been corrected

Line 208: Please be consistent in your word choices. On line 208 you use “pre-school centres” which I believe you defined as “pre-primary schools” on lines 205-6. I apologize if these two school types are different, but if they are different then please define “pre-school centres” alongside pre-primary schools.

**Author’s response:** Terms homogenized

Lines 367-8: In Figure 1’s caption: “Nevertheless, the role of the size of the bubble group seems relevant only in scenarios of high transmission rate.” I do not see any data or plots related to bubble group size in this figure. This may be due to mixing up the order of the Figures when they were uploaded or mixing up the order of the figure captions since Figure caption 2 seems to correspond to Figure 1 and vice-versa.

**Author’s response:** Thank you for spotting this. There was a mistake while uploading and numbering the figures. Figures 1 and 2 were incorrectly labeled. We have relabeled them and reuploaded all figures in the correct order.

Figures 4 & 5 appear to be missing captions in the body of the text making it hard to interpret the plots. I found the captions later in the manuscript and now see that the authors used MSD to determine the “best” R* for primary and secondary schools. Based on the curves, I think it would be worth commenting in the manuscript that the “best” R* is good at predicting a low number of secondary infections but are very poor at estimating a higher number of secondary infections (i.e., at 9 secondary infections an R* closer to 0.9 is better. Overall Figure 4 looks to busy with axis labels blending into different subplots, please clean this up.

**Author’s response:** We have “cleaned” Figure 4 according to the reviewer’s suggestion. We have also moved forward the captions just after the conclusions of the manuscript to facilitate the search. We agree with the reviewer that the numerical curves do not correctly fit the data for large outbreaks, actually, we had already discussed such disagreement in the previous version, see lines [471-473], however, we have also introduced some text in the lines [466] following the suggestion of the reviewer.

Lines 531-2: The authors state that “schools were a key containment measure tool.” This statement seems to suggest that if children did not go to school then transmission in the community would be higher. I am not sure that the work in this manuscript shows that to be the case. I do however agree with the statement that schools “facilitated epidemiological surveillance.” Later in the paragraph the authors continue to state that schools contain the virus, which I do not believe is proven in this manuscript. However, the authors are correct to state that R* is likely to be well below 1 and thus schools are not likely to result in outbreaks. Again, all this analysis needs to be in the context of pre-delta variant since schools in the US did have outbreaks amongst the students when the delta variant was present in Fall 2021. The authors do mention at the end of the discussion the context of alpha and delta variants as well as vaccinations. However, I feel it would be a stronger manuscript if the context of when this study was conducted receives more emphasis. One solution would be to change verb tenses from the present to the past when discussing the results since this will imply to the reader that the findings in this manuscript may no longer apply to the current time in the pandemic.

**Author’s response:** We have included more references to the pre-Delta variant era throughout the manuscript. We have toned down the assertions that did not seem proven by the analysis. We have changed the verb tenses also, as suggested.

Overall, I find this to be an important piece of work that will add to the discussion of whether or not to close schools during this pandemic and future pandemics. This manuscript shows that having in-person education may not result in large outbreaks for some diseases, such as COVID-19 pre-variants. These findings will serve as important information for any future scientific and policy discussions.

**Author’s response:** Thank you for this constructive thought.

_____________________________________________________________________________

**Reviewer #2**

Reviewer #2: The authors have made some changes in the writing of the paper and corrected some of the errors and inconsistencies pointed by the different referees. For instance, they have published the code of the simulation in a public repository. Some other questions, like the definition of outbreak throughout the paper or the full description of the dataset have not been resolved. The article needs to be compared with current evidence on COVID-19 and school opening. The authors still describe their analysis as "modelling exercises" (L347).

In my previous report, I recommended rejection because, in my opinion, the conclusions of the paper are not supported by the data or the analysis. There is no statistical analysis which can measure the association or causality of school opening on incidence. In order to present conclusions like "SARS-CoV-2 infections and COVID-19 cases detected in Catalan schools appear to closely mirror the underlying community transmission from the neighbourhoods where they are set" the authors need to present a framework in which the question of association can be considered.

**Author’s response:** We thank the reviewer for the insightful comments, but again, we must state that we respectfully disagree with them. To start with, we are indeed presenting models fitted to the available data, so the terminology we have used remains, in our opinion valid. Second, we do strongly believe that the conclusions of the paper are indeed supported by the data and the analysis, particularly after the changes we made from the initial submission, following the advice of both reviewers. Finally, our data seem to align with current evidence from other contexts like Japan (Fukumoto K, McClean CT, Nakagawa K. No causal effect of school closures in Japan on the spread of COVID-19 in spring 2020. Nat Med. 2021 Oct 27. doi: 10.1038/s41591-021-01571-8.) and USA (Ertem Z, Schechter-Perkins EM, Oster E, van den Berg P, Epshtein I, Chaiyakunapruk N, Wilson FA, Perencevich E, Pettey WBP, Branch-Elliman W, Nelson RE. The impact of school opening model on SARS-CoV-2 community incidence and mortality. Nat Med. 2021 Oct 27. doi: 10.1038/s41591-021-01563-8.), and with other studies about the role of schools in Catalonia during the 2020-2021 academic course (Perramon A. et al; Schools as a Framework for COVID-19 Epidemiological Surveillance of Children in Catalonia, Spain: A Population-Based Study. Front Pediatr 2021 Vol. 9 Pages 754-744; DOI: 10.3389/fped.2021.754744).
