# Supplementary material for: Individual prevention and containment measures in schools in Catalonia, Spain, and community transmission of SARS-CoV-2 after school re-opening
Source: PLoS One. 2022 Feb 16;17(2):e0263741. doi: 10.1371/journal.pone.0263741 (PMC8849486; doi:10.1371/journal.pone.0263741)
Supplement: Attachment — Submitted filename: Response 3 to Reviewers_FINAL.docx [file pone.0263741.s007.docx]

**Third response to reviewers**

**PONE-D-21-21706-R2**

**Manuscript title**: "Individual prevention and containment measures in schools in Catalonia, Spain, and impact of this strategy on community transmission of SARS-CoV-2 after school re-opening"

_____________________________________________________________________________

REVIEWER COMMENTS

**6. Review Comments to the Author**

**Reviewer #4:**

Since I join the review process from the current version, I do not intend to delay it. Thus all my comments are suggestive, not mandatory; I just want to improve the manuscript.
**Author’s response:** We do appreciate this statement and predisposition to help and offer constructive criticism. We have tried to address all comments which did not imply a substantial rewriting or redesigning of the manuscript.

I do not understand why the paper examines a wide range of R* values. It seems that the authors only have to focus on the estimated values, 0.35 and 0.55. Otherwise, I am curious why they choose the specific values of 0.3, 0.6, …, and 1.5 (p. 14).

**Author’s response:** We wanted to explore all the possible scenarios and compare the current data with different opinions on the propagation of the SARS-CoV-2 in the schools. Some researchers (actually some of the reviewers) consider that schools act as triggers of the spreading of the epidemics and therefore, seems plausible to consider large R* values, at least above 1. In this way we can clearly show the expected dynamics from our numerical simulations and the clear disagreement with the actual data.

I believe there remains much room to improve the way the paper presents in its figures. For instance, Figure 1 displays bar graphs where my eyes focus on differences among R* values, though the authors seem to call attention to variation between bubble size (p. 12). An alternative is line graphs. Similar comments hold for Figure 2 as well.

**Author’s response:** We thank the reviewer for this comment, but in the previous three versions of the manuscript we already had to change the figures on various occasions. We would prefer to maintain the figures as they are.

I am afraid some notations lack explanation, so that it is not easy to read.

- On p. 11, I do not understand why the paper calculates “the homogenized cumulative incidence” as they do. Why does it multiply the cumulative incidence by the positivity rate? And then why does it divide it by a baseline? Why is the baseline 5%?

**Author’s response:** We used the 5% baseline because this is the threshold provided by WHO as an indicator for the good level of testing and control. Nevertheless, we use for comparison the ratio between each age group and the population. Therefore, the 5% disappears. Given your concerns and those of other reviewers, we have decided to define directly the ratio between homogenized incidences which was, in fact, the indicator that is shown in the heatmap and discussed in the results. We did not aim to develop a method for assessing the real incidence, but for better comparing the incidence of different age groups with regards to the average one and taking into account the diagnostic effort.

- On p. 12, R^X and N_index suddenly appear without explanation. I can imagine what they mean, though I would like to see their definition.

**Author’s response:** In the previous version of the manuscript we had written: “we defined R*as the effective reproduction number inside the bubble groups.” in our opinion was already a definition, however, as it is not clear enough we have rewritten as: “*we defined R* as the effective reproduction number, or average number of children infected by the index case, inside the bubble groups*.” And after the formula “*where N_index corresponds to the total number of index cases found.*”

- In Figure 4, what does “SI” stand for? Secondary infection or single infected kids?

**Author’s response:** The reviewer is right, it corresponds to secondary infection. We have modified the figure because of a previous comment of another reviewer and did not change the caption. From “*Fraction of groups going into quarantine with a number N of infected kids by COVID-19 for primary (A) and secondary (B) schools*” we change to “*Fraction of groups going into quarantine with a number of secondary infected (SI) kids by COVID-19 for primary (A) and secondary (B) schools*”

Accordingly, I do not understand Figures 4A and 4B. By the way, the range of R* values considered is 0.15 to 0.9, which is different from those of Figures 1 and 2. Why?

**Author’s response:** In this case we do not want to show the possible values of R* as in Figure 1 and 2, but the vicinity values of R* to show the almost linear relationship in panels E and F and the minimal value around 0.35 and 0.55 in panels C and D

- In particular, I recommend not using the word “bubble” (without definition) in the abstract. My sense is that it is not such a common usage.

**Author’s response:** We would prefer to maintain the terminology which has been used throughout the past months here in Spain, and which has become the preferred terminology when referring to “stable groups of children doing activities together” (and with clear role in tracing and epidemiological purposes). We hope this is found acceptable.

**Reviewer #5:** 
“Individual prevention and containment measures…” is a major article that uses mathematical modeling informed by real-world data to estimate 1) the R* in school settings following initial re-opening during the 2020-21 school year in Catalonia, and 2) to estimate the impact of two specific strategies (quarantine duration and bubble size) on in-school transmission. Similar to other studies, the authors find that the R* in the school setting was low (although this finding was previously published by some of the same authors in Pediatric Infectious Diseases Journal: <https://journals.lww.com/pidj/Fulltext/2021/11000/Age_dependency_of_the_Propagation_Rate_of.2.aspx>). Despite the study’s title, the authors did not really measure the impact of school opening on community transmission, and any mention of the impact of school opening on community transmission should be removed from the title, abstract, etc, and moved to the discussion/background sections. Other recently published manuscripts have addressed this question (e.g., Ertem et al, Nature Medicine: <https://www.nature.com/articles/s41591-021-01563-8>).

**Author’s response:** We have removed the concept of impact from the title, which now reads *“Individual prevention and containment measures in schools in Catalonia, Spain, and community transmission of SARS-CoV-2 after school re-opening”*. We have modified our running head to “Reopening of schools and overall SARS-CoV-2 community transmission”. We have also modified the abstract following the reviewer’s suggestion. However, after various backs and forwards with previous reviewers, we prefer to maintain the rest of the manuscript mostly as it was after revision 2.

Throughout the manuscript, methods and results are intermixed. These sections need to be completely re-organized and re-written, such that methods are clear and that the results follow from the methods described. In addition, the authors need to be clear about what they did and did not study – specifically, they modeled quarantine and bubble size as infection control strategies. Those should be the focus of all aspects of the manuscript – the rest does not follow from their results.

**Author’s response:** This is partly the result of responding to previous queries raised after the original manuscript and with additional iterations in revision 1 and 2. At this stage, we kindly request that we do not have to rewrite the manuscript.

Specific Comments:
Abstract:
Methods/results not clear and are not organized. Please restructure.

**Author’s response:** See previous response

Conclusions: Please remove any discussion of the impact of schools on community transmission. That was not studied in this manuscript (in addition, a simple correlation is not sufficient to evaluate this question, as the impact of a policy change is not seen in incidence rates for 3-6 weeks. Community incidence rates reflect new introductions into schools, and then a lag period is needed to see the impact of school opening on community transmission- this would not be expected to occur instantaneously, but rather with a lag which is not addressed in the abstract or the manuscript).

**Author’s response:** We have tried to modify this as requested.

Author Summary
-Clarify if surveillance was mandatory

**Author’s response:** Yes, surveillance was mandatory. We have added this to the author’s summary.

-In the second bullet, clarify that the mathematical model used real-world inputs

**Author’s response:** Done

-In the final bullet, add “pre-vaccine” in addition to “pre-delta”

**Author’s response:** Done

-In the what do these findings mean? Section, remove sections on impact of school opening on community transmission (first bullet) and the bullet on closing schools as a last resort (last bullet)– their study provides no insight into this question

**Author’s response:** Done

Manuscript
-Lines 170-171: Rephrase to “the extent to which children of varying ages can be effective drievrs of supershedding events” for clarity

**Author’s response:** Done

-Lines 175-179 – It is not clear that schools closed pre-maturely – early in the pandemic, very little was known. The decision for school closures was based on models of influenza, which may have been appropriate early, even if we learned otherwise later.

**Author’s response:** We agree, and we have replaced the word “prematurely” by “rapidly”.

-Lines 190-191 – add recent publications about impact of school mode on community transmission. There are several papers published on this topic (See Ertem et al).

**Author’s response:** Thank you for this suggestion. We have included this recent reference.

Methods/Results
-See major comment above. These sections need to be entirely reorganized and re-written such that there is a clear methods section and a clear results section. Currently, these are entirely intermingled.

**Author’s response:** We kindly disagree and we would prefer to maintain the current structure which is the result of two previous interactions with reviewers.

-The entire section is highly technical and difficult to understand for those without a statistical background. Strongly recommend re-writing such that this is more understandable for a more general scientific audience.

**Author’s response:** Again, we prefer to maintain the section as it is. The models are not necessarily easy to understand but it was important that they are well described.

-Lines 495 – 525 – Authors appear to have used a simple correlation to evaluate impact of schools on community rates, however, this method is fundamentally not a valid analysis. Schools exist as part of a community infrastructure, such that a case from the community is introduced into a school, where it can spread, and then that second case can spread to others in the community, which can then lead to more introductions into the school setting and the potential for more spread. However, this process takes time. There is a lag between a primary and a secondary case, such that a week to week correlation is not the appropriate measure for identifying the impact of school opening on community spread – a window period needs to be considered. This section could be rephrased to simply state that cases in schools tended to be lower than cases in the community at the same point in time, and any discussion of the impact of schools on community rates removed, as the authors did not evaluate this question.

**Author’s response:** We have addressed this comment and toned down our conclusions in relation to impact I transmission. The impact of the schools on the community can be addressed from the point of view that for each “event” 1 index case enters from the community to the bubble group, and after the spreading only 0.35 or 0.55 cases goes to the community. In this sense it is stated that the impact of bubble groups in schools is low.

Discussion
-As noted repeatedly above, the authors did not study the impact of schools on community transmission, but rather modeled transmission in schools (which the authors previously published) and the impact of specific infection control measures in school settings on in-school transmission. This should be the focus of all of their statements about what they did and did not find.

**Author’s response:** We have addressed this comment and toned down our conclusions in relation to impact on transmission.

-While it is accurate that the study was conducted pre-delta, it was also conducted pre-vaccine, and both developments are likely to impact the findings in unpredictable ways. Both should be discussed/acknowledged.

**Author’s response:** We agree, and have added the following: “*Similarly, this study was conducted prior to the vaccination of teenagers or school children, and further research will be needed to understand the positive additional impact that vaccination may have in intra-school transmission.*”

**Reviewer #6:** 
I think this paper has some valuable material and provides interesting results about school transmission, but there are some problems that make it still fairly far from acceptable for PLOS One at this time.

A lot of it is just very unclear. For example, CI_hom is introduced and I could not figure out what the motivation was. Why not just use incidence? Reading further, I gather that they want to factor in imperfect ascertainment. This was not mentioned earlier, and even assuming that I don’t understand the mystery factor of 0.05. There are lots of things like this.

**Author’s response:**  We used the 5% baseline because this is the threshold provided by WHO as an indicator for the good level of testing and control. Nevertheless, we use for comparison the ratio between each age group and the population. Therefore, the 5% disappears. Given your concerns and those of other reviewers, we have decided to define directly the ratio between homogenized incidences which was, in fact, the indicator that is shown in the heatmap and discussed in the results. We did not aim to develop a method for assessing the real incidence, but for better comparing the incidence of different age groups with regards to the average one and taking into account the diagnostic effort.

There is some tone that doesn’t belong in a scientific paper in my opinion. I agree with the authors that school closures for COVID-19 were not the best policy (with the benefit of hindsight) but there are a few spots which I detail below where they take this for granted, whereas the job of the paper is to persuade the reader of this.

**Author’s response:** We have tried to tone down our conclusions and make them more evidence-based.

From reading the abstract I thought they were going to just estimate the number of transmissions from each index case by counting the number of cases in a cluster and subtracting one. Maybe factoring in imperfect ascertainment would be good, but the surveillance seems pretty good. However, as I got into the paper there is a model that is much more complicated and the R* is estimated by tweaking parameters in the model and fitting to data. This is fine, but I would like this to be explained better.

**Author’s response:** The study proposed by the reviewer “counting the number of cases in a cluster and subtracting one” actually corresponds to equation in page 12, we did for all the index cases and therefore, we calculated the average. We do not focus on this because such study was done in parallel and is already published by some of us, see Alonso et al 2021, ref 18.

However, the reviewer is right because it is not correctly stated in the abstract. Therefore, we have modified the abstract. We have previously “*The effective reproduction number inside the bubble groups in schools (R*), defined as the average number of schoolmates infected by each primary case within the bubble, was calculated, yielding a value of 0.35 for primary schools and 0.55 for secondary schools*,” and we have in the new version: “*The effective reproduction number inside the bubble groups in schools (R*), defined as the average number of schoolmates infected by each primary case within the bubble, was calculated, yielding a value of 0.35 for primary schools and 0.55 for secondary schools, and compared with the outcomes of the mathematical model*”

Page 4, I didn’t understand the sentence on lines 93 to 97
“the proportion of “bubble groups” (stable groups of children doing activities together) confined ranged” So only 1 to 5% of all students were in a bubble group? Or bubble groups each had between 1 and 5 students? And I don’t see the connection with the other parts of the sentence. I think “confined ranged” is maybe a typo.

**Author’s response:** We have rephrased this for clarity to “*During the entire first term, the proportion of “bubble groups” (stable groups of children doing activities together) that were forced to undergo confinement ranged between 1 and 5%*...”

Line 98 “the effect of different parameters part of the defined preventive strategies” I couldn’t parse this.

**Author’s response:** We have rephrased this to “*Mathematical models were also used to evaluate the effect of different parameters related to the defined preventive strategies (size of the bubble group, number of days of confinement required by contacts of an index case).”*

Line 104 “Homogenized monthly cumulative incidence (CIhom) was assessed to compare the epidemiological” I don’t know what homogenized monthly cumulative incidence is. Can you rephrase in some way to make it clearer what you’re doing? Also, what is the subject of the verb “suggested”? The CI_hom? Does it suggest something? Or does your analysis suggest it?
**Author’s response:** This has been rewritten, as explained in a previous comment. We have also corrected the “suggested”.

Page 6, line 124. I think the phrase “extraordinarily infrequent” is too strong. (My numbers are around 1 in 200 children with COVID infection are hospitalized.

**Author’s response:** We have removed the “extraordinarily”

Page 8, line 175. The word “premature” contains a value judgement that has not been justified yet. You could say it was used prematurely.

**Author’s response:** This has been amended.

Line 189, “re-state” Not the right word. “Reignite’?

**Author’s response:** This has been corrected as suggested.

Lines 191 to 192. “but again, in the context of insufficient evidence of the real contribution of children and schools to ongoing transmission” I find the tone here inappropriate. This is a scientific paper, not a blog post or an editorial page. The authors’ job (if they want to persuade the reader) is to provide evidence that schools are safe and that closures are a bad idea. Saying that there was insufficient evidence to close schools with a new variant is a value judgement. I don’t think it belongs in the introduction. You can summarize a situation and the decisions made without broadcasting your judgment in every sentence.

**Author’s response:** We have removed the last sentence so as to be more objective and less opinionated.

Page 9, line 196-197. This is not a sentence, but a fragment. (No predicate.) Same with the following sentence.

**Author’s response:** These two sentences have been rewritten.

Page 10, line 225. Suggest replacing “agile” with “rapid”.

**Author’s response:** Done

Page 10-11. I do not understand the purpose of “homogenized cumulative incidence”. Firstly, would cumulative incidence just be the total number of detected cases in a time interval? Secondly, what is the positivity rate? The rate at which PCR tests come back positive? Why would I multiply that by incidence? And divide by 0.05?

**Author’s response:**  This has been rewritten, as explained in a previous comment.

Page 11 “Although it does not aim to evaluate the real incidence” Why is it better than CI_j?

**Author’s response:** This has been rewritten, as explained in a previous comment.

Page 12, line 291 “kid” is informal in English. Use “child” or “student”

**Author’s response:** We have substituted all references to “kids”

Line 315, do you mean the superscript to be x in this equation?

**Author’s response:** The superscript has to be *. There were some problems with the format, and therefore it is still X, however the reviewer is right.

Page 13, The section heading say “Statistical Methods” it starts with talking about bubbles. I don’t see any statistical method discussed here.

**Author’s response:** We have performed “Monte Carlo” Simulations for the different scenarios described in the manuscript. However, we partially agree that such methods may be not considered “statistical methods” but “mathematical tools for the comparison” of the average value with the data. We have added this information under the original “mathematical model” section, ad removed the “statistical methods” section.

Page 15. I’m somewhat skeptical of the good fit in Figure 3, especially given that there were no free parameters. Or did the authors choose the parameters like 0.35 to get a good fit? If they did, that would be fine, but they should be transparent about it. I agree that the number of cases in schools appears to track the number in the community very closely.

**Author’s response:** We have employed the factor 0.35 and it was the only fit parameter in figure 3. However, such a parameter is not completely free, because we have a justification of this particular value in lines 410-413. There is freedom in the argument and close values (0.30-0.40) may be also justifiable too, and in this sense it is a fit. We have modified the caption to make it clearer: “*Comparison with the estimations proportional to the 7-days moving average of the 10-day cumulative incidence in Catalonia using (1-𝜂)𝜉=0.35 (A10, green solid line),*”

Page 18. I didn’t find this to be a compelling analysis. I actually agree that school reopenings were unlikely to have a huge effect on community transmission, but this is not systematic investigation. What are they estimating exactly? I think the thing that is persuasive is just that there is apparently not much transmission in schools. You can’t infer much from one single reopening event in one jurisdiction where you observe what happens to incidence.

**Author’s response:** We have toned down our assertion that school transmission did not play a role in community transmission, and focused on the fact that intra-school transmission was limited.

Page 22, “presential” is not a commonly used English word. How about “present” or “current”.

**Author’s response:** We have substituted “presential” by “face-to-face”

”Keeping schools open is allowing children to benefit from presential schooling, a fundamental right that should not be questioned unless stronger evidence emerges proving the contrary” Again, I agree, but stating that something is a right is an ethical statement, not a scientific one.

**Author’s response:** Here we kindly disagree with the reviewer’s views, given that education is a fundamental human right, which was jeopardized by school closures. We would kindly request that our concluding thought can be maintained.
